# Histone modifications in laryngeal squamous cell carcinoma: from epigenetic reprogramming to precision therapy

**DOI:** 10.3389/fimmu.2026.1981328

**Published:** 2026-10-08

**Authors:** Zexi Lv, Qiuping Li, Xiaodong Feng

**Affiliations:** 1 Department of Rehabilitation Center, The First Affiliated Hospital of Henan University of Chinese Medicine, Zhengzhou, China; 2 Department of Rehabilitation Medicine, Henan University of Chinese Medicine, Zhengzhou, China; 3 Department of Traditional Chinese Medicine, Guangdong Provincial People’s Hospital (Guangdong Academy of Medical Sciences), Southern Medical University, Guangzhou, Guangdong, China

**Keywords:** aryngeal squamous cell carcinoma, epigenetics, histone acetylation, histone lactylation, histone methylation, histone modification, therapeutic resistance

## Abstract

Laryngeal squamous cell carcinoma (LSCC) is a common malignancy of the head and neck, accounting for the vast majority of laryngeal cancers. Its development involves complex interactions between genetic and epigenetic alterations. Histone post-translational modifications, as a crucial regulatory layer linking intracellular metabolic states, oncogenic signaling, and transcriptional programs, participate in tumor cell proliferation, epithelial-mesenchymal transition, stemness maintenance, metabolic reprogramming, and therapeutic resistance by modulating chromatin conformation, transcription factor accessibility, and gene expression profiles. This study systematically summarizes recent advances in histone acetylation, methylation, lactylation, and other novel histone modifications in LSCC, further discusses crosstalk among different histone modifications and their potential roles in the tumor microenvironment and resistance to radiotherapy, chemotherapy, and immunotherapy, and analyzes therapeutic strategies targeting histone modifications as well as future prospects for single-cell and spatial epigenomics in precision medicine for LSCC, aiming to provide new theoretical foundations for biomarker development and epigenetic-targeted therapies in LSCC.

## 1 Introduction

Laryngeal squamous cell carcinoma (LSCC) is a major anatomical subtype of head and neck squamous cell carcinoma (HNSCC) and the most common histological type of laryngeal malignancy. According to GLOBOCAN 2022 data, approximately 189, 000 new cases of laryngeal cancer and 103, 000 related deaths occur globally each year, indicating its substantial global disease burden (1). Smoking and alcohol consumption are the most well-established risk factors for LSCC (2). Clinical manifestations vary significantly across different subsites of the larynx, with some patients presenting at locally advanced stages at diagnosis. Disease control and survival outcomes remain poor for locally advanced, recurrent, or metastatic cases (3). Currently, surgery, radiotherapy, and platinum-based chemotherapy remain the mainstay treatments for LSCC. In recent years, immune checkpoint inhibitors have further transformed the treatment landscape for recurrent or metastatic HNSCC; however, long-term benefits are still limited to a subset of patients (4, 5).

Tumor initiation and progression are not solely determined by changes in DNA sequences. Epigenetic mechanisms—including DNA methylation, histone modifications, chromatin remodeling, and non-coding RNAs—can continuously reshape gene expression programs without altering the DNA sequence, collectively contributing to malignant transformation, tumor progression, and therapeutic resistance (6–9). Compared with relatively fixed DNA sequence alterations, many epigenetic states are dynamic and potentially reversible. This characteristic not only enables them to participate in transitions of tumor cell states but also makes chromatin-modifying enzymes and their associated pathways attractive targets for drug intervention (6, 10). The fundamental structural unit of chromatin is the nucleosome, composed of an octamer of histones H2A, H2B, H3, and H4, around which DNA is wrapped (11). Multiple amino acid residues on the N-terminal tails and globular domains of histones can undergo various post-translational modifications (PTMs), including acetylation, methylation, phosphorylation, ubiquitination, as well as lactylation, crotonylation, and succinylation (12–15). These modifications collectively shape chromatin structure and transcriptional programs by modulating nucleosome stability and histone-DNA interactions, creating specific binding sites for reader proteins, and regulating the recruitment of chromatin remodeling complexes and transcription factors (12).

In recent years, comprehensive reviews have systematically synthesized the roles of histone modifications across HNSCC as a heterogeneous disease entity (16, 17). Nevertheless, substantial inter-subtype molecular heterogeneity—encompassing somatic mutational landscapes, transcriptional regulatory networks, DNA methylation profiles, tumor hypoxia gradients, and immune microenvironment composition—has been well documented among anatomically distinct HNSCC subsites (18). Consequently, mechanistic insights derived from pan-HNSCC cohorts may not be fully generalizable to LSCC, a biologically and clinically unique subtype. Moreover, LSCC-specific evidence on histone modifications remains fragmented and undercharacterized, particularly concerning rapidly evolving metabolism-linked modifications—including lysine lactylation—which are expanding the conceptual and functional scope of epigenetic regulation beyond classical paradigms.Building on existing knowledge from HNSCC, this review provides an LSCC-centered synthesis of histone acetylation, methylation, lactylation, and other emerging histone modifications, with emphasis on their roles in tumor progression, metabolic reprogramming, immune regulation, and therapeutic resistance program (**Figure 1**).

**Figure 1:**
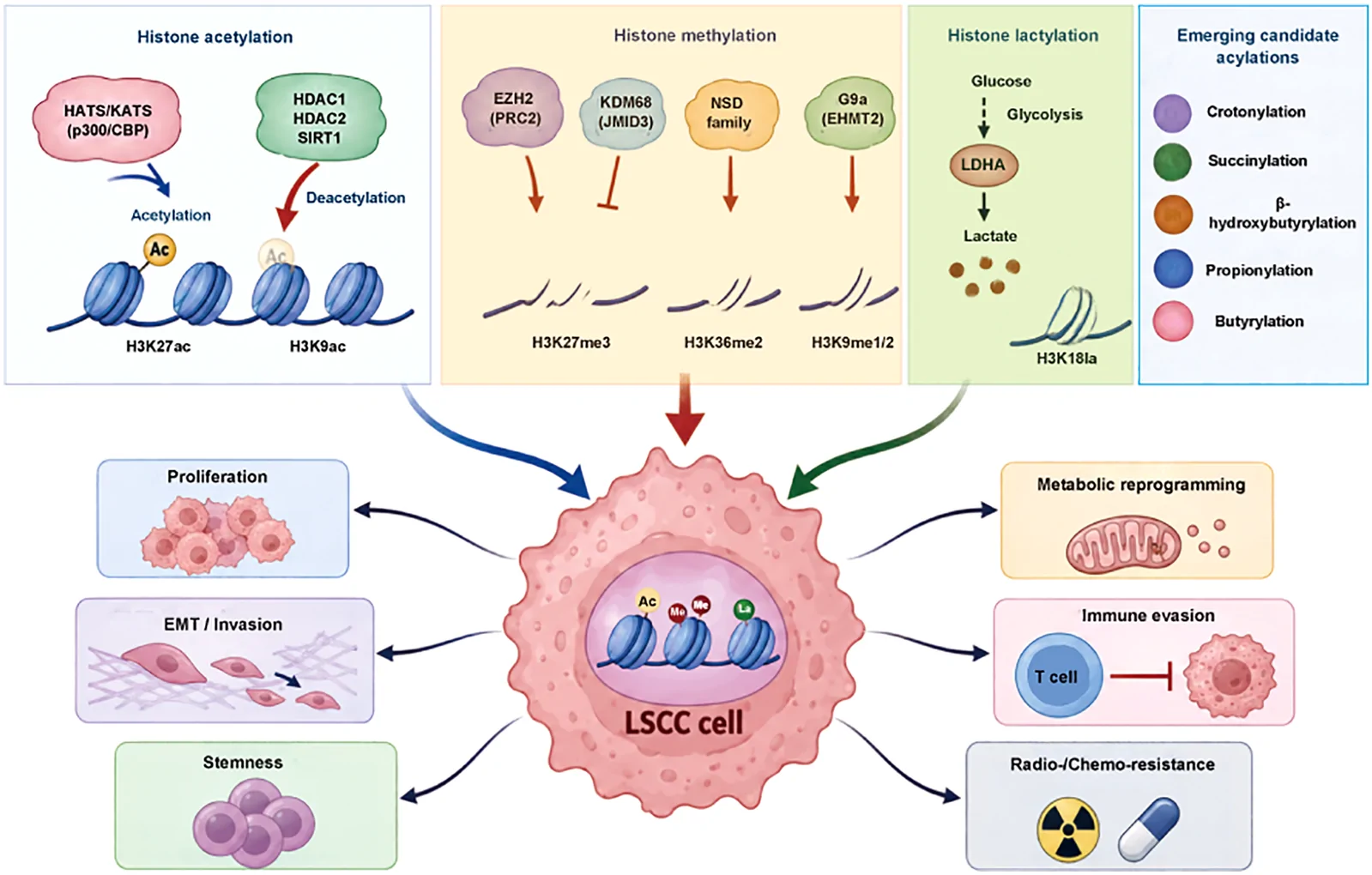
Histone modification in LSCC. Overview of the principal histone-modification programs implicated in laryngeal squamous cell carcinoma (LSCC), including acetylation/deacetylation, methylation, lactylation, and emerging acylations, together with their major biological consequences.

## 2 Histone modification and its regulatory system

Histone modifications represent a highly dynamic epigenetic regulatory process, primarily maintained by three classes of molecules: “writers”, “erasers”, and “readers.” Writers add chemical groups to specific histone residues, including histone acetyltransferases (KATs/HATs) and lysine methyltransferases (KMTs/HMTs); erasers remove these modifications, such as histone deacetylases (HDACs) and lysine demethylases (KDMs); readers recognize specific histone marks via distinct structural domains and recruit transcription factors, chromatin remodeling complexes, or other effector proteins, thereby translating these chemical marks into functional chromatin states and transcriptional outcomes (12, 19, 20). Different histone modifications exhibit clear site- and context-specificity: H3K27ac typically marks active enhancers, H3K4me3 is predominantly enriched at active promoters, and H3K9ac generally correlates with transcriptionally active open chromatin. In contrast, H3K27me3 is mainly involved in polycomb-mediated transcriptional repression, while H3K9me3 is closely associated with heterochromatin formation and stable gene silencing (21–24). However, individual histone marks are usually insufficient to determine the final transcriptional state of genes; instead, different modifications can interact through synergy, competition, or antagonism, and are interpreted by specific readers into diverse biological effects, thus forming a dynamic “histone code” that depends on cellular and chromatin contexts (12, 25, 26). This system also extensively crosstalk with DNA methylation and ATP-dependent chromatin remodeling processes, collectively shaping cell-type-specific chromatin states and transcriptional programs. Given the pronounced intratumoral and intertumoral heterogeneity characteristic of LSCC, a systems-level interrogation of the cooperative and dynamic interactions among multiple histone modifications—and their integrated regulatory networks—holds promise for elucidating the epigenetic mechanisms that govern the establishment and maintenance of functionally distinct malignant cell states.

## 3 The role of histone acetylation/deacetylation in LSCC

Histone acetylation is a reversible epigenetic modification catalyzed by histone acetyltransferases (HATs, such as p300/CBP, GCN5, Tip60, etc.), which neutralizes the positive charge of lysine residues, thereby relaxing chromatin structure and promoting transcriptional activation. The reverse process is mediated by HDACs. Acetylation typically neutralizes lysine’s positive charge, loosening chromatin structure (promoting transcription), whereas deacetylation restores the positive charge, making chromatin more condensed (inhibiting transcription). In LSCC, the imbalance between acetylation and deacetylation is primarily reflected in the overexpression of HDACs, although genome-wide studies targeting specific acetylation sites remain relatively limited.

HDACs are a class of important epigenetic regulatory enzymes responsible for removing acetyl groups from lysine residues on the N-termini of histones (mainly H3 and H4). The human genome encodes 18 HDACs, which are generally classified into four classes based on sequence homology to yeast orthologs and catalytic properties (27, 28). Class I HDACs (HDAC1, HDAC2, HDAC3, HDAC8) are highly homologous to yeast Rpd3, predominantly localized in the nucleus, and often serve as catalytic subunits of co-repressor complexes such as Sin3, NuRD, and CoREST, playing broad roles in gene transcription repression, cell cycle regulation, cellular differentiation, and embryonic development (29, 30). Class II HDACs are further subdivided into class IIa (HDAC4, HDAC5, HDAC7, HDAC9) and class IIb (HDAC6, HDAC10); these enzymes can shuttle between the nucleus and cytoplasm, exhibiting broader substrate specificity and greater flexibility in cellular localization (27). Class III HDACs, known as sirtuins (SIRT1–7), require NAD^+^ as a cofactor for activity and are closely associated with cellular energy metabolism, redox homeostasis, and aging (31). Class IV contains only HDAC11, which possesses structural features shared by both class I and class II HDACs (32). Differences among HDAC types in subcellular localization, substrate preference, and regulatory networks determine their distinct biological functions in cancers such as LSCC. Among them, HDAC1 is the most extensively studied member of class I; it catalyzes the deacetylation of core histones (H2A, H2B, H3, H4) and also targets non-histone substrates (such as p53, STAT3, NF-κB, SP1), participating in transcriptional regulation, DNA repair, cell cycle progression, and apoptosis (29, 33, 34).

Zhao R et al. conducted immunohistochemical analysis on 90 LSCC tissue samples, 30 adjacent non-tumor tissues, and 30 laryngeal polyp tissues, revealing that HDAC1 was significantly upregulated in LSCC tissues, primarily localized in epithelial cells. Its expression levels were positively correlated with T stage, lymph node metastasis, tumor location, and clinical stage, and were higher in patients with low radiosensitivity, suggesting its potential as a marker for poor prognosis and radioresistance (35). Ding et al. recently delineated a mechanistic role for HDAC1 in mediating chemoresistance to 5-fluorouracil (5-FU) and cisplatin in LSCC. Their findings demonstrate that combined 5-FU and cisplatin treatment induces HDAC1 upregulation in LSCC cells, with significantly elevated HDAC1 expression observed in acquired chemoresistant sublines. Genetic knockdown of HDAC1 markedly restored cellular sensitivity to both agents. Mechanistically, HDAC1 promotes chemoresistance through dual transcriptional and post-transcriptional regulation of interleukin-8 (IL-8): it directly binds the IL-8 promoter to enhance transcriptional activation, while concurrently repressing miR-93 expression—leading to diminished miR-93–mediated degradation of IL-8 mRNA and consequent stabilization of IL-8 protein. Pharmacological or genetic inhibition of HDAC1 effectively reverses this axis and restores chemosensitivity *in vitro* and *in vivo* (36). An early study reported that HDAC2 was highly expressed in HEp-2 cells and that its downregulation inhibited proliferation and migration, induced apoptosis, reduced MMP2/9 expression, and increased caspase-3 and caspase-9 cleavage (37). However, HEp-2 cells were subsequently recognized as HeLa-contaminated and are therefore not considered an authentic LSCC model; consequently, these findings should be interpreted with caution (37). SIRT1 shows reduced expression in laryngeal and hypopharyngeal cancers, which is significantly associated with tumor staging and lymph node metastasis (38). Additionally, Vaiciulis P et al. investigated single-nucleotide polymorphisms (SNPs) of SIRT1 and found that carriers of the rs3758391 T/T genotype exhibit a statistically significant increase in LSCC susceptibility. Moreover, individuals harboring the rs3818292 AG genotype displayed significantly lower serum SIRT1 concentrations compared with GG homozygotes, suggesting a potential genotype–phenotype relationship. However, neither rs3758391 nor rs3818292 was associated with 5-year overall survival in LSCC patients after multivariable adjustment (39). Notably, Uloza et al. failed to replicate an association between the SIRT1 rs12778366 polymorphism and LSCC risk in an independent cohort, highlighting inter-population heterogeneity in SIRT1 genetic effects (40).

In addition to HDAC-mediated deacetylation, increasing evidence supports a role for histone acetyltransferase/co-activator pathways in LSCC (41, 42). P300 is overexpressed in LSCC tissues and has been associated with adverse clinicopathological features. More recently, CBP/EP300-mediated transcriptional activation of PFKL was shown to promote glycolysis, lactate accumulation and cisplatin resistance in LSCC, accompanied by alterations in H3K27ac and H3K9ac. Nevertheless, compared with HDAC1, the site-specific histone acetylation landscape and direct chromatin targets of p300/CBP in LSCC remain insufficiently characterized. Overall, acetylation imbalance leads to silencing of tumor suppressor genes and activation of oncogenes (43), and is involved in epithelial-mesenchymal transition (EMT) and maintenance of cancer stem cells.

Histone methylation in LSCC: the EZH2/H3K27me3 axis and emerging H3K36 methylation programsHistone methylation is an important class of post-translational modification that primarily occurs on lysine (K) or arginine (R) residues of core histones, especially H3 and H4, forming mono-, di-, or trimethylation states (me1, me2, me3) (44, 45). This modification is catalyzed by histone methyltransferases (HMTs/KMTs, known as “writers”), using S-adenosylmethionine as the methyl donor (46, 47), while its removal is carried out by histone demethylases (KDMs, referred to as “erasers”). Proteins that recognize specific methylated marks are termed “readers, “ which can translate these modifications into downstream chromatin remodeling or transcriptional effects (48, 49). Unlike acetylation, which generally promotes chromatin openness, the biological functions of histone methylation depend on the specific residue and degree of methylation.

Enhancer of Zeste Homolog 2 (EZH2) is the core catalytic subunit of the Polycomb repressive complex 2 (PRC2), primarily mediating transcriptional repression through the trimethylation of lysine 27 on histone H3 (H3K27me3). It is one of the most extensively studied and representative histone methyltransferases in LSCC (50). Studies have shown that EZH2 is significantly overexpressed in LSCC tissues and closely associated with malignant clinicopathological features. Elevated EZH2 expression correlates strongly with advanced T stage, poor pathological differentiation, and increased risk of lymph node metastasis, and serves as an independent prognostic factor for LSCC patients (51, 52). Mechanistically, Luo H et al. demonstrated that EZH2 promotes EMT via H3K27me3-dependent epigenetic regulation, thereby enhancing the invasive and metastatic potential of LSCC cells. Furthermore, functional studies revealed that EZH2 overexpression not only promotes LSCC cell proliferation and progression through the cell cycle but also enhances tumor-forming capacity and stemness characteristics *in vivo* (53, 54), suggesting that EZH2-mediated H3K27me3 remodeling may simultaneously contribute to tumor growth, metastasis, and maintenance of cancer stemness. Given that cancer stem cells typically exhibit enhanced DNA damage repair, anti-apoptotic activity, and treatment resistance, EZH2-driven epigenetic programs may further promote the survival of residual LSCC tumor cells, contributing to therapeutic resistance and tumor recurrence. Overall, the EZH2/H3K27me3 axis appears to play a pivotal role across multiple critical stages of LSCC progression—including proliferation, EMT, metastasis, stemness acquisition, and potential drug resistance—positioning it as a key epigenetic regulatory hub linking malignant progression and therapeutic resistance in LSCC.

In contrast to EZH2-mediated H3K27 methylation, lysine-specific demethylase 6B (KDM6B), also known as Jumonji domain-containing protein 3 (JMJD3), catalyzes the demethylation of H3K27me3/2, thereby epigenetically counteracting EZH2/PRC2-mediated transcriptional repression. Zhang W et al. found that aberrant expression of JMJD3 is closely associated with poor prognosis in HNSCC (55), suggesting that the dynamic balance between H3K27 methylation and demethylation may contribute to the malignant progression of HNSCC. Beyond H3K27 modification, methylation regulators targeting other lysine residues are also gaining increasing attention. For instance, members of the NSD family are major regulators of H3K36 methylation, particularly H3K36me2, whereas G9a (EHMT2) primarily catalyzes H3K9 mono- and dimethylation (H3K9me1/2). Studies have shown that these methyltransferases can influence tumor cell stemness, immune microenvironment, and related transcriptional programs, thereby contributing to HNSCC progression (56, 57). In particular, NSD1 inactivation leads to reduced H3K36me2 levels, accompanied by redistribution or increased H3K27me3, further promoting an “immunocold” tumor phenotype characterized by low immune cell infiltration, which may impair antitumor immune responses and treatment sensitivity (58, 59). These findings indicate that histone methylation regulation in HNSCC is not confined to a single EZH2/H3K27me3 axis but involves a complex epigenetic network composed of multiple methylation marks—including H3K27, H3K36, and H3K9—and their respective regulatory enzymes. However, direct studies on the expression patterns, molecular mechanisms, and clinical significance of KDM6B/JMJD3, the NSD family, and G9a in LSCC remain limited. Therefore, while existing research on HNSCC provides important clues for understanding potential histone methylation abnormalities in LSCC, whether these regulatory mechanisms can be directly extrapolated to LSCC requires further validation.

## 4 Histone lactylation driven by metabolic reprogramming

Tumor cells often exhibit enhanced glycolysis, known as the Warburg effect, even under relatively sufficient oxygen supply. In this process, glucose is metabolized via glycolysis to produce pyruvate, which is further converted into lactate by lactate dehydrogenase A (LDHA) (60). The discovery of histone lactylation has expanded the biological significance of lactate, transforming it from a traditional end product of glycolysis into a key mediator linking tumor metabolic reprogramming with epigenetic regulation. In hypoxic and highly glycolytic tumor regions, accumulated lactate not only acidifies the tumor microenvironment, thereby affecting immune and stromal cell functions, but also provides a metabolic substrate for protein lactylation, influencing chromatin state and gene transcription (61). Similar to other malignancies, LSCC also undergoes significant metabolic reprogramming that contributes to tumorigenesis and progression (62). Studies have further shown that multiple molecular pathways promote enhanced glycolysis and lactate accumulation in LSCC cells (63, 64), suggesting that the “glycolysis/lactate accumulation—histone lactylation—transcriptional reprogramming” axis may serve as an important epigenetic bridge connecting metabolic abnormalities to stable malignant phenotypes. Daidi Fu et al. found significantly elevated levels of lysine 18 lactylation on histone H3 (H3K18la) in LSCC tissues; inhibition of lactate production led to a corresponding decrease in H3K18la levels, accompanied by reduced tumor cell proliferation and migration, as well as increased apoptosis. Mechanistic studies revealed that H3K18la promotes RUNX2 expression, subsequently activating the PI3K/AKT signaling pathway and enhancing invasive phenotypes in LSCC. Inhibition of lactate production and associated lactylation levels using sodium oxalate or similar approaches partially reversed these malignant traits (65). Furthermore, Liu et al. demonstrated that gene signatures related to lactate metabolism effectively predict prognosis in LSCC patients (66), further highlighting the close association between lactate metabolic dysregulation and LSCC biology and clinical outcomes. Overall, existing evidence supports that lactate is not merely a byproduct of metabolic reprogramming in LSCC, but may also convert metabolic states into persistent pro-tumorigenic transcriptional programs through epigenetic modifications such as H3K18la, thereby driving LSCC cell proliferation, migration, and invasion, providing potential therapeutic targets for targeting lactate metabolism and histone lactylation.

## 5 Other novel histone modifications

In addition to acetylation, methylation, and lactylation, histones can undergo various acyl modifications, including crotonylation, succinylation, β-hydroxybutyrylation, propionylation, and butyrylation. Since the acyl donors required for these modifications are closely linked to metabolic intermediates such as crotonyl-CoA, succinyl-CoA, β-hydroxybutyrate, and propionyl-CoA, they serve as critical interfaces connecting cellular metabolic status with chromatin transcriptional regulation (67–70). Histone crotonylation is generally enriched at active promoters and enhancers; p300/CBP can utilize crotonyl-CoA to catalyze histone crotonylation, while HDAC1/2/3 possess de-crotonylation activity, enabling dynamic regulation of this modification (13, 71–73). Notably, the transcriptional effects of crotonylation are site- and chromatin context-dependent; for example, H3K27cr can also mediate transcriptional repression via the GAS41/SIN3A-HDAC1 complex (74). Histone succinylation is closely associated with succinyl-CoA and the state of the TCA cycle; abnormalities in the TCA cycle can lead to redistribution of chromatin succinylation accompanied by transcriptional changes, while SIRT5 exerts NAD^+^-dependent de-succinylation activity (15, 75–77). Given the prominent metabolic reprogramming observed in LSCC, alterations in the abundance and compartmentalization of metabolites and acyl-CoA donors could theoretically reshape histone crotonylation, succinylation, and other metabolically coupled acylationsprogram (62–64), However, unlike H3K18 lactylation, direct evidence linking these modifications to LSCC initiation or progression is currently lacking. Accordingly, histone crotonylation, succinylation, β-hydroxybutyrylation, propionylation, and butyrylation should presently be regarded as candidate metabolic–epigenetic mechanisms rather than established drivers of LSCC. Future studies combining histone PTM profiling with CUT&Tag and transcriptomic analyses may help determine whether specific acylation marks have functional or clinical relevance in LSCC.

To facilitate comparison of the available evidence, the major histone modifications, regulatory enzymes, biological functions, experimental models, and potential therapeutic implications reported in LSCC and related HNSCC studies are summarized in **Table 1**.

**Table 1:** Major histone modifications and regulatory enzymes implicated in LSCC and related HNSCC studies.

Histone modification/regulator	Major molecular mechanism or downstream pathway	Reported biological effects	Evidence model/source	LSCC-specific evidence	Potential therapeutic/clinical implication	Ref.
HDAC1/histone deacetylation	Histone and non-histone deacetylation; regulation of IL-8 at transcriptional and post-transcriptional levels; potential involvement in DNA damage repair	Tumor progression, lymph-node metastasis, radioresistance and resistance to 5-FU/cisplatin	LSCC patient tissues; LSCC cell models; *in vivo* models	Yes – relatively strong	HDAC1 may serve as a prognostic/radiosensitivity biomarker; HDAC inhibition may enhance chemotherapy or radiotherapy response	(35, 36)
HDAC2/histone deacetylation	Regulation of proliferation-, migration- and apoptosis-related programs; effects on MMP2/9 and caspase signaling	Reduced proliferation and migration and increased apoptosis following HDAC2 suppression	HEp-2 cell model	Limited/uncertain, because HEp-2 is HeLa-contaminated and is not considered an authentic LSCC model	Biological relevance requires revalidation in authenticated LSCC models before therapeutic interpretation	(37, 107)
SIRT1/NAD+-dependent deacetylation	NAD+-dependent deacetylation linking epigenetic regulation with cellular metabolism and stress responses	Associations with tumor stage, lymph-node metastasis and LSCC susceptibility; inconsistent genetic associations across populations	LSCC/hypopharyngeal carcinoma tissues; LSCC patient genetic association studies	Yes, mainly clinical-association evidence	Potential biomarker value, although prognostic and causal relevance remains uncertain	(38–40)
p300/CBP/histone acetylation	Lysine acetyltransferase activity and transcriptional co-activation of oncogenic programs	Promotion of oncogenic transcription and tumor growth	Mainly HNSCC/OSCC preclinical models	No direct robust LSCC evidence	KAT inhibition represents a potential strategy but requires LSCC-specific validation	(41, 42)
EZH2/H3K27me3	PRC2-mediated H3K27 trimethylation; repression of target genes; regulation of EMT, stemness and cellular plasticity	Proliferation, invasion, metastasis, EMT, stemness, poor differentiation and unfavorable prognosis	LSCC patient tissues; LSCC cell and *in vivo* models	Yes – strong LSCC-specific evidence	One of the most promising epigenetic targets in LSCC; EZH2 inhibitors may potentially improve treatment sensitivity	(50–54)
KDM6B/JMJD3/H3K27 demethylation	Removal of H3K27me3/2 and functional counterbalance to EZH2/PRC2-mediated repression	Regulation of malignant transcriptional programs and therapeutic vulnerability	Primarily HNSCC models	No direct established LSCC evidence	Potential therapeutic relevance through modulation of the H3K27 methylation-demethylation balance	(55)
NSD family/H3K36 methylation	Regulation of H3K36 methylation, particularly H3K36me2; crosstalk with H3K27me3	Maintenance of epigenomic state, tumor-cell programs and immune phenotype	Primarily HNSCC/squamous-cell carcinoma models	No direct established LSCC evidence	May define epigenetic subgroups and therapeutic vulnerabilities; requires LSCC-specific validation	(56–59)
NSD1 loss/reduced H3K36me2	Reduced H3K36me2 with redistribution/increase of H3K27me3; suppression of CXCL9/CXCL10	T-cell exclusion, ‘immune-cold’ phenotype and impaired antitumor immunity	HNSCC and squamous-cell carcinoma models	No direct LSCC-specific validation	May contribute to resistance to immune checkpoint blockade and potentially identify epigenetically immune-cold tumors	(58, 59)
G9a/EHMT2/H3K9me1/2	H3K9 mono- and dimethylation-mediated transcriptional repression	Regulation of stemness and malignant transcriptional programs	Mainly HNSCC and other cancer models	Limited/indirect	Potential epigenetic therapeutic target; its role in LSCC remains to be established	(56, 57)
H3K18la/histone lactylation	Lactate-dependent H3K18 lactylation activates RUNX2, subsequently enhancing PI3K/AKT signaling	Increased proliferation, migration and invasion; decreased apoptosis	LSCC tissues and LSCC experimental models	Yes – direct LSCC evidence	Provides a direct metabolic–epigenetic therapeutic axis; inhibition of lactate production/lactylation may suppress malignant phenotypes	(65)
Lactate metabolism/LDHA-related lactylation	Enhanced glycolysis and lactate accumulation provide a metabolic environment permissive for histone lactylation	Metabolic reprogramming, tumor progression and potentially persistent oncogenic transcriptional states	LSCC metabolic studies plus LSCC H3K18la evidence	Partially supported in LSCC	Targeting glycolysis, LDHA or lactate-dependent epigenetic regulation represents a potential therapeutic strategy	(62–66)
H3K18la-associated immune regulation	In other cancers, H3K18la can promote PD-L1-related transcriptional programs and impair CD8+ T-cell activity	Immune suppression and reduced response to immune checkpoint blockade	NSCLC and other non-LSCC cancers	No direct LSCC evidence	Provides a hypothesis-generating mechanism linking LSCC lactate metabolism to immunotherapy resistance; requires direct validation	(91–93)
H3K27ac/histone acetylation	Active enhancer-associated chromatin; regulation of immune-modulatory gene transcription including PD-L1-related programs	Transcriptional activation and modulation of tumor immunogenicity	Mainly HNSCC models	Indirect evidence for LSCC	May contribute to immune-response heterogeneity and represent a target for HDAC/KAT-based combination therapy	(21, 82)
H3K27me3-mediated immune regulation	EZH2-dependent repression of antigen-presentation programs, including MHC-I-related pathways	Reduced antigen presentation, impaired CD8+ T-cell response and anti-PD-1 resistance	HNSCC experimental models	Not yet specifically established in LSCC	Provides rationale for combining EZH2 inhibition with immune checkpoint blockade	(81)
Histone crotonylation	p300/CBP-mediated crotonylation; HDAC1/2/3-mediated decrotonylation; chromatin-context-dependent transcriptional regulation	Regulation of active or, at selected sites, repressive transcriptional states	General molecular studies/non-LSCC models	No direct LSCC evidence	Candidate metabolic–epigenetic mechanism that should be investigated by histone PTMomics and CUT&Tag in LSCC	(13, 71–74)
Histone succinylation	Succinyl-CoA-dependent histone modification; SIRT5-mediated desuccinylation; linkage to TCA-cycle metabolism	Metabolic–chromatin coupling and transcriptional regulation	General molecular studies/non-LSCC models	No direct LSCC evidence	Potential link between mitochondrial metabolic abnormalities and chromatin regulation in LSCC; currently hypothesis-generating	(15, 75–77, 115)
Other metabolic histone acylations (β-hydroxybutyrylation, propionylation, butyrylation)	Histone acylation driven by corresponding metabolic intermediates/acyl-CoA species	Metabolism-dependent regulation of chromatin and transcription	General epigenetic/metabolic studies	No direct LSCC evidence	Emerging candidate marks for unbiased histone PTM profiling in LSCC	(67–70)

LSCC, laryngeal squamous cell carcinoma; HNSCC, head and neck squamous cell carcinoma; OSCC, oral squamous cell carcinoma; HDAC, histone deacetylase; SIRT1, sirtuin 1; EZH2, enhancer of zeste homolog 2; PRC2, polycomb repressive complex 2; KDM6B, lysine demethylase 6B; JMJD3, Jumonji domain-containing protein 3; NSD, nuclear receptor-binding SET domain; EHMT2, euchromatic histone lysine methyltransferase 2; LDHA, lactate dehydrogenase A; ICI, immune checkpoint inhibitor; PTM, post-translational modification.

## 6 Crosstalk between different histone modifications: remodeling the “histone code” of LSCC

The same lysine site can undergo different modifications: H3K27 can form either H3K27ac (active enhancer/promoter) or H3K27me3 (polycomb repression), and the dynamic balance between these two states may constitute an epigenetic switch that determines transcriptional status. H3K18 can be either acetylated (H3K18ac) or lactylated (H3K18la); when cells shift to a high-glycolytic, high-lactate state, the ratio of these modifications may redistribute, necessitating comparison of the H3K18la/H3K18ac ratio and its corresponding transcriptional programs. Furthermore, H2AK119ub and H3K27me3 exhibit Polycomb synergy, while phosphorylation, acetylation, and methylation collectively regulate chromatin structure following DNA damage. The true epigenetic state of LSCC should thus be understood as a dynamic network composed of multiple coexisting modifications.

## 7 Histone modification and the tumor microenvironment of LSCC

The tumor microenvironment consists of tumor cells, immune cells, fibroblasts, endothelial cells, and extracellular matrix. Different cell types exhibit distinct metabolic activities and chromatin states, making it difficult to determine the source of elevated modifications in the overall tissue. Hypoxia is a key factor linking the microenvironment to histone modifications: HIF-1α drives glycolysis and lactate accumulation, promoting lactylation (78), while simultaneously altering the functions of enzymes such as HDACs and KDMs, thereby affecting acetylation and methylation (79, 80). LSCC-specific studies have documented alterations in PD-L1 expression and immune-cell infiltration, including T cells and macrophage populations. However, direct mechanistic evidence linking specific histone modifications to MHC-I antigen presentation, CXCL9/CXCL10 expression, or PD-L1 regulation in LSCC remains limited. Current mechanistic evidence for these epigenetic–immune connections is derived predominantly from broader HNSCC cohorts and other tumor types and therefore requires validation in LSCC.methprogram (81, 82). Current studies on HNSCC suggest that epigenetic drugs can alter tumor immunogenicity and enhance the efficacy of immune checkpoint inhibitors; however, research specifically focusing on the “histone modification–immune microenvironment” axis in LSCC remains in its early stages (**Figure 2**).

**Figure 2:**
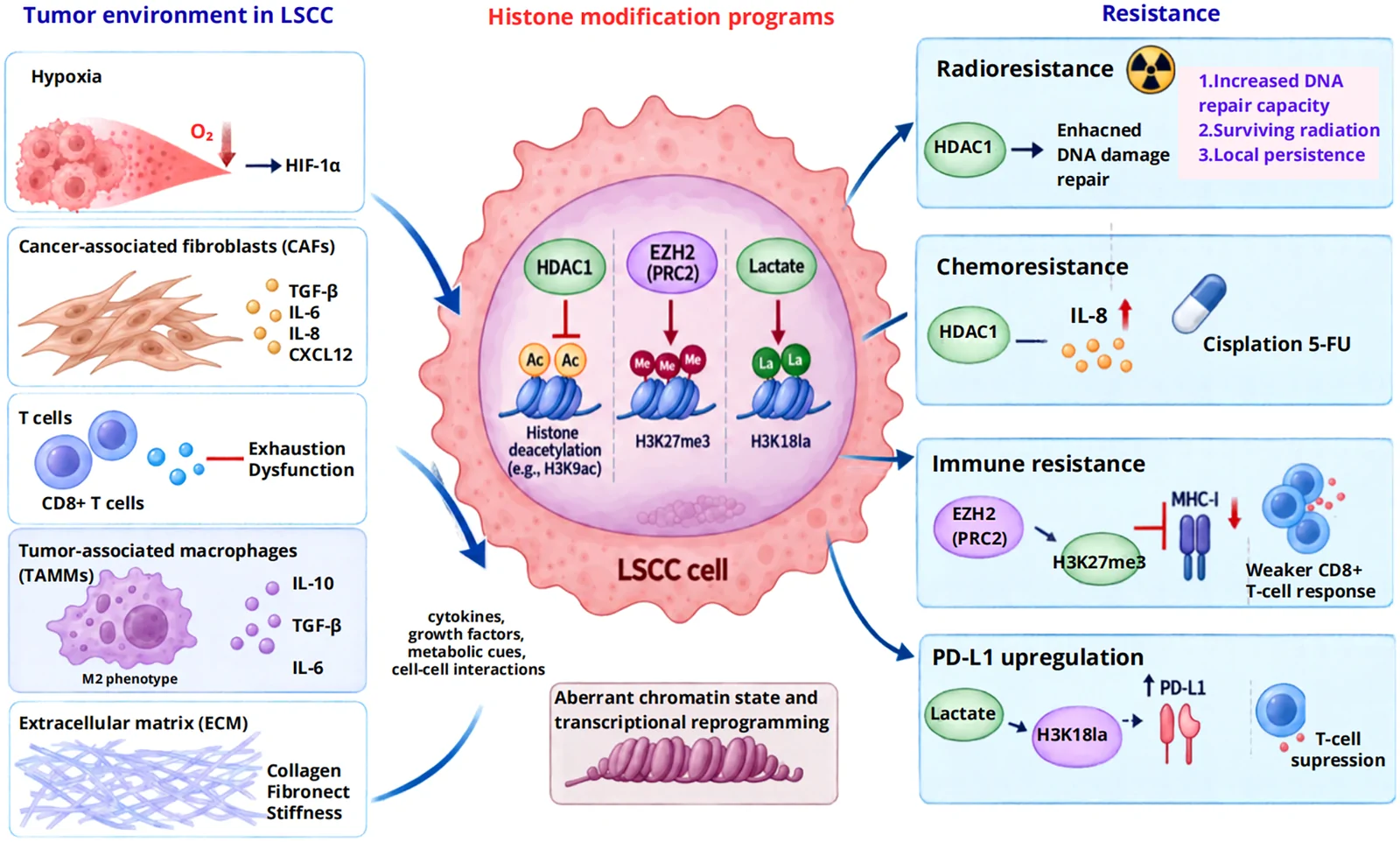
Histone modification, tumor microenvironment, and therapy resistance in LSCC. Integrative model linking the tumor microenvironment, histone-modification programs, and treatment resistance in LSCC. Evidence for radioresistance and chemoresistance is based on LSCC studies, whereas the antigen-presentation axis is extrapolated from HNSCC, and the PD-L1/lactylation immune-resistance axis remains hypothetical in LSCC.

## 8 Histone modification and resistance to LSCC treatment

High expression of HDAC1 is associated with low radiosensitivity and poor survival in LSCC (35), providing relatively direct evidence that histone modifications are involved in clinical radiotherapy response. The underlying mechanism may involve enhanced DNA damage repair capacity, abnormal chromatin state, and dysregulated cell cycle control. Jin Ho Kim et al. demonstrated that specific knockdown of HDAC1 increases the radiosensitivity of SQ20B cells, accompanied by persistent γ-H2AX foci after irradiation, indicating delayed repair of DNA double-strand breaks (83). Future studies could further validate the mechanistic framework that “aberrant HDAC or EZH2 activity alters chromatin state, thereby enhancing DNA damage repair capacity and ultimately leading to radioresistance, “ through systematic evaluation combining γ-H2AX, RAD51, and markers related to homologous recombination and non-homologous end joining (84). The HDAC1/IL-8 axis has been shown to promote resistance of LSCC to 5-fluorouracil and cisplatin, while combining HDAC inhibitors with cisplatin enhances the antitumor effects in certain laryngeal cancer cells (36). Furthermore, EZH2-mediated maintenance of stemness and cellular plasticity may further promote the survival of residual tumor cells after chemotherapy. Studies on HNSCC have revealed that EZH2 is significantly enriched in cisplatin-resistant cells and cancer stem cell populations, and can activate the Wnt/β-catenin signaling pathway by suppressing adenomatous polyposis coli transcription via H3K27me3, thereby maintaining the cancer stem cell phenotype and reducing cisplatin sensitivity. Inhibition of EZH2 reduces the proportion of cancer stem cells and restores cisplatin sensitivity in resistant cells (85). Additionally, EZH2-driven EMT and phenotypic plasticity are also associated with decreased cisplatin sensitivity in HNSCC (86). These findings suggest that EZH2-mediated epigenetic plasticity may provide a cellular basis for residual disease and subsequent recurrence by sustaining treatment-resistant stem-like subpopulations.

Immune checkpoint inhibitors (ICIs) have become a crucial therapeutic approach for recurrent or metastatic HNSCC, yet only a subset of patients achieve durable clinical benefit, indicating significant heterogeneity in treatment response (4, 5). Growing evidence suggests that aberrant chromatin states may influence immunotherapy outcomes by modulating tumor immunogenicity and immune cell recruitment. In HNSCC models rather than LSCC-specific systems, EZH2-mediated H3K27me3 suppresses MHC-I antigen presentation, and EZH2 inhibition enhances CD8^+^ T cell responses and reverses anti-PD-1 resistance in experimental models (81). Similarly, NSD1 inactivation leads to reduced H3K36me2 and increased H3K27me3, which suppresses CXCL9/CXCL10 expression, promotes T cell exclusion, and correlates with resistance to PD-1 blockade (59). Histone acetylation status also plays a role in the transcriptional regulation of immune-modulatory molecules such as PD-L1 in HNSCC (82). Although these findings provide a mechanistic framework potentially relevant to LSCC, their applicability to LSCC requires direct experimental and clinical validation. Based on these mechanisms, combination therapies involving HDAC and EZH2 inhibitors with immune checkpoint blockade have entered early clinical investigation in HNSCC (87, 88). However, these trials have not established LSCC-specific efficacy, and histone-targeted precision therapy in LSCC remains largely at the preclinical or hypothesis-generating stage. Future studies will require LSCC-specific molecular stratification and prospective validation to identify patients most likely to benefit from epigenetic therapies.On the other hand, lactate produced via glycolysis can itself shape an immunosuppressive microenvironment by impairing effector functions of T/NK cells and promoting the formation of immunosuppressive myeloid cells (89, 90). Further studies reveal that LDHA-driven lactate production and MCT-mediated lactate transport not only sustain lactate accumulation in the tumor microenvironment but may also convert metabolic abnormalities into persistent immunosuppressive transcriptional programs through histone lactylation. For instance, H3K18la promotes PD-L1 expression via the POM121/MYC axis and weakens CD8+T cell cytotoxicity, while inhibiting glycolysis/lactylation enhances anti-PD-1 therapy efficacy (91). Lactate-induced histone lactylation also increases IL-10 expression and immunosuppressive function in tumor-associated macrophages (92), and has been linked to resistance to immune checkpoint inhibitors in other cancers (93). program Taken together, these observations from other malignancies support a hypothetical metabolic–epigenetic model in which an LDHA/MCT–lactate–histone lactylation axis may contribute to immunosuppression and ICI resistance. However, this pathway has not yet been directly established in LSCC, and its relevance to LSCC immune escape and immunotherapy resistance requires dedicated mechanistic and clinical validation (**Figure 2**).

## 9 New technologies advance research on LSCC histone modifications

Traditional Western blot and immunohistochemistry mainly reflect the overall levels and tissue distribution of specific histone modifications, but are limited in resolving their genome-wide localization and direct target genes. ChIP-seq has greatly advanced genome-wide studies of histone modifications; however, conventional protocols typically require substantial starting material, restricting their application in limited clinical samples and rare cell populations (94). In contrast, CUT&RUN and CUT&Tag demand less input material and offer higher signal-to-noise ratios, enabling chromatin analysis even at extremely low cell numbers or single-cell resolution (95, 96), making them particularly suitable for epigenomic studies using LSCC biopsies and limited clinical specimens. These technologies can be systematically employed to map the genome-wide distribution of key histone modifications such as H3K27ac, H3K27me3, H3K4me3, and H3K18la. Notably, H3K18la CUT&Tag has been used to identify lactylation-associated target genes, and recent studies in OSCC have further demonstrated the feasibility of combining H3K18la CUT&Tag with RNA-seq to pinpoint direct lactylation targets (97). Integrating CUT&Tag with RNA-seq and ATAC-seq holds promise for establishing a complete regulatory pathway linking changes in histone modifications, chromatin accessibility remodeling, and transcriptional alterations in target genes (98).

At higher resolution, single-cell ATAC-seq can reveal chromatin accessibility differences among distinct cell populations in LSCC, while scCUT&Tag enables direct profiling of cell-type-specific histone modification states such as H3K27ac, H3K27me3, and H3K4me3 (99, 100). These technologies could be used in the future to investigate whether H3K18la is preferentially enriched in highly glycolytic tumor cells, tumor-associated macrophages, or cancer-associated fibroblasts, as well as whether H3K27me3 remodeling is concentrated in treatment-resistant tumor cell subpopulations. Meanwhile, spatial-CUT&Tag and integrated spatial epigenomics-transcriptomics approaches have already enabled mapping of histone modifications—including H3K27ac, H3K27me3, and H3K4me3—while preserving tissue architecture (101, 102), laying a technical foundation for further exploration of epigenetic heterogeneity within LSCC tumor nests, invasive fronts, and different regions of the immune microenvironment. On the other hand, mass spectrometry-based histone PTMomics allows for relatively unbiased identification and quantification of multiple histone PTMs, including methylation, acetylation, lactylation, crotonylation, and succinylation (13, 14, 103), and with advances in low-input mass spectrometry, it is becoming increasingly feasible for application to limited clinical samples (104). Collectively, these technologies provide an opportunity to establish a stepwise multi-omic framework for LSCC histone modification research. Histone PTM profiling could first be used to identify aberrant modification sites in patient samples, followed by CUT&Tag to define their genomic localization, ATAC-seq to assess associated chromatin accessibility, and bulk or single-cell RNA sequencing to determine transcriptional consequences. Single-cell and spatial epigenomic approaches could subsequently resolve the cell-type and spatial specificity of these alterations, while functional perturbation and independent clinical cohorts would be required to establish causality and clinical relevance. Such an integrated workflow may advance LSCC research from isolated histone-marker observations toward a spatially and functionally resolved histone PTM landscape.analysetoward (**Figure 3**).

**Figure 3:**
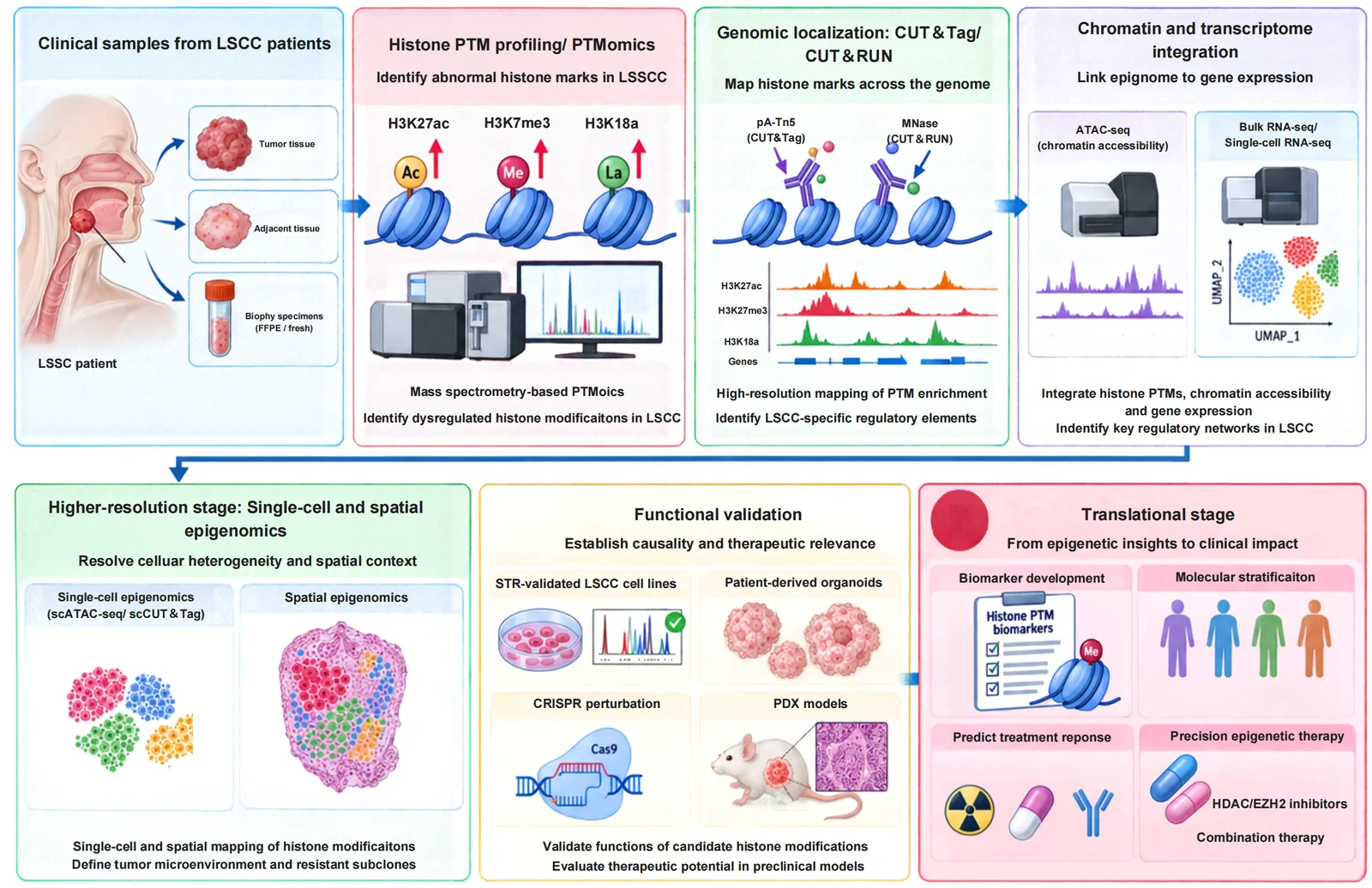
Identify abnormal histone marks in LSCC. Proposed multi-omics roadmap for future histone-modification studies in LSCC, spanning clinical sample acquisition, histone PTM discovery, genomic mapping, transcriptomic integration, single-cell/spatial epigenomics, functional validation, and translational development.

## 10 Current issues and future perspectives

Although research on histone modifications in LSCC has made some progress, several critical issues remain to be addressed. First, evidence specifically associated with LSCC is still relatively limited, and much of the mechanistic understanding comes from mixed HNSCC cohorts. However, HNSCCs arising from different anatomical subsites exhibit significant differences in genetic mutations, transcriptional profiles, DNA methylation patterns, hypoxia status, and tumor immune microenvironments; thus, findings from pan-HNSCC studies cannot be directly applied to LSCC (18, 105, 106). Second, some early studies of laryngeal cancer employed HEp-2 cells, which were subsequently shown to be HeLa-contaminated and therefore do not represent an authentic LSCC model (107). Therefore, future studies should prioritize using authentic LSCC cell lines verified by STR analysis, combined with patient-derived organoids, primary tumor cells, and PDX models, to enhance disease specificity and clinical relevance (108–110). Currently, research on histone modifications in LSCC remains largely focused on modification enzymes such as HDAC and EZH2, along with a few individual histone marks, while comprehensive genomic mapping and functional integration studies targeting key modifications like H3K27ac, H3K27me3, and H3K18ac are still notably insufficient (16, 17, 111). Future research should also expand beyond single modifications to explore crosstalk among different histone modifications—for example, the well-known antagonistic relationship between H3K27ac and H3K27me3 (112), as well as potential competitive modification and dynamic balance between H3K18ac and H3K18la under different metabolic states (113). Meanwhile, the coupling between metabolism and epigenetics deserves further attention: acetyl-CoA and succinyl-CoA serve as acyl donors for histone acylation reactions, while α-ketoglutarate and NAD^+^ participate in the catalytic processes of JmjC family demethylases and sirtuin deacetylases, enabling metabolic status to directly influence chromatin structure and transcriptional programs (114, 115). Ultimately, efforts should shift histone modification research from mechanistic discovery toward clinical stratification by integrating information from histopathology, transcriptomics, proteomics, metabolomics, and epigenomics to establish LSCC-specific epigenetic subtypes, thereby exploring their predictive value for patient prognosis, radiosensitivity, cisplatin sensitivity, and response to immunotherapy (116, 117). Similar integrative strategies combining inflammatory-metabolic biomarkers with routine clinical variables have shown the feasibility of improving risk stratification and assessing model transportability through external validation in other otolaryngology-related diseases (118).

## 11 Conclusion

Histone modifications represent a crucial epigenetic regulatory layer linking genetic background, oncogenic signaling, metabolic status, and the tumor microenvironment. Current studies have implicated several histone-modifying regulators, particularly HDAC1 and EZH2, in LSCC progression, while evidence for other regulators remains less extensive or derives partly from preclinical and non-LSCC models. Altered histone acetylation and H3K27 methylation have been associated with tumor-cell proliferation, epithelial–mesenchymal transition, stemness, and therapeutic resistance. Recently, the discovery of the H3K18la-RUNX2-PI3K/AKT axis further demonstrates that lactate metabolism can directly reshape the transcriptional program in LSCC through histone lactylation, establishing a novel mechanistic intersection between metabolic reprogramming and epigenetic regulation. However, current studies in this field remain largely focused on single modification enzyme expression analysis and *in vitro* cellular experiments, lacking comprehensive histone modification maps with spatial and cell-type resolution. Future integration of CUT&Tag, mass spectrometry-based PTMomics, single-cell, and spatial epigenomics technologies to dissect crosstalk among different histone modifications, along with systematic exploration of combination strategies involving HDAC inhibitors, EZH2 inhibitors, and the lactate-lactylation axis with radiotherapy, chemotherapy, and immunotherapy, holds promise for advancing LSCC research from descriptive epigenetics toward mechanism-driven, therapy-oriented precision epigenetics.
